# Gamete Cryopreservation: Molecular Mechanisms of Cellular Injury and Epigenomic Alterations in Human and Animal Models

**DOI:** 10.3390/ijms27219468

**Published:** 2026-10-23

**Authors:** Xinxin Zhu, Plamen Todorov, Lora Gaydarova, Volodimir Isachenko

**Affiliations:** 1 Department of Obstetrics and Gynecology, Medical Faculty, Cologne University, 50931 Cologne, Germany; xinxinzhu96@gmail.com; 2 Institute of Biology and Immunology of Reproduction, Bulgarian Academy of Sciences (BAS), 1113 Sofia, Bulgaria; plamen.ivf@gmail.com (P.T.); lora_gaydarova@abv.bg (L.G.)

**Keywords:** gamete cryopreservation, spermatozoa, oocytes, cryoinjury mechanisms, mitochondrial dysfunction, oxidative stress, ferroptosis, epigenomic regulation

## Abstract

In human spermatozoa, freezing and thawing are consistently associated with impaired membrane integrity, reduced motility, mitochondrial dysfunction, oxidative imbalance and alterations in DNA/chromatin stability. Regulated cell death pathways may additionally contribute to post-thaw deterioration; however, the strength of evidence differs among individual pathways, and emerging mechanisms such as ferroptosis remain less extensively characterized in human spermatozoa than conventional oxidative and apoptosis-associated injury. Evidence for cryopreservation-associated epigenomic alterations is similarly heterogeneous. Experimental and animal studies demonstrate changes in DNA methylation, histone modifications and non-coding RNA profiles. In contrast, human evidence is generally more reassuring and has not demonstrated consistent persistent abnormalities in major imprinting-associated methylation patterns. Accordingly, observations from animal models should not be directly extrapolated to human reproductive outcomes. By integrating human and experimental evidence while explicitly considering these differences in evidentiary strength, this review identifies established mechanisms, emerging molecular pathways and major unresolved questions in contemporary gamete cryobiology.

## 1. Introduction

Cryopreservation has become an essential component of assisted reproductive technology (ART), enabling long-term preservation of spermatozoa and oocytes for medical and non-medical indications. The principal approaches include slow programmable freezing and vitrification, which differ in their cooling conditions, cryoprotectant requirements and mechanisms of ice formation [1,2,3].

Slow programmable freezing employs controlled cooling rates to regulate extracellular ice formation and promote progressive cellular dehydration, thereby reducing the probability of intracellular ice formation and excessive osmotic injury. In contrast, vitrification combines rapid cooling with relatively high cryoprotectant concentrations to achieve a highly viscous, glass-like state while minimizing crystalline ice formation. Both approaches require careful control of cryoprotectant exposure, cooling and warming conditions to balance protection against ice-related injury with the risks of osmotic and chemical stress [2,3].

Despite substantial technical progress, cryopreservation may induce structural, functional and molecular alterations in gametes. These include membrane destabilization, osmotic imbalance, oxidative stress, mitochondrial dysfunction, DNA damage and disturbances of intracellular regulatory mechanisms. The biological consequences depend strongly on gamete-specific characteristics. Spermatozoa are particularly susceptible to membrane and acrosomal injury, loss of motility, mitochondrial dysfunction and alterations in chromatin integrity. Oocytes, because of their larger size, high water content and complex cytoskeletal organization, are additionally vulnerable to meiotic spindle disruption, intracellular calcium imbalance and disturbances of cellular volume regulation [1,4].

Recent investigations have extended the assessment of cryoinjury beyond conventional morphological and functional parameters to include DNA methylation, histone modifications and non-coding RNA regulation [5]. However, the biological significance of these alterations remains incompletely understood, particularly because findings obtained in animal models cannot always be directly extrapolated to human gametes.

This narrative review critically examines the cellular and molecular mechanisms of cryopreservation-induced injury in human spermatozoa and oocytes, integrating complementary evidence from animal and experimental models. Particular attention is given to the relationships between oxidative stress, mitochondrial dysfunction, regulated cell death and epigenomic alterations, as well as their potential implications for fertilization, embryonic development and offspring health.

## 2. Literature Search Strategy

This article was designed as a narrative review supported by a structured literature search rather than as a systematic review or meta-analysis. The purpose of the search was to identify representative and mechanistically informative studies addressing cellular, molecular and epigenomic consequences of gamete cryopreservation, with particular emphasis on human spermatozoa and complementary evidence from human oocytes and experimental animal models.

The literature search was performed using PubMed and Google Scholar and included peer-reviewed publications available up to August 2026. Search terms were used individually and in different combinations and included ‘human’, ‘mammalian’, ‘sperm’, ‘spermatozoa’, ‘cryopreservation’, ‘freezing’, ‘thawing’, ‘cryodamage’, ‘cryoprotective agents’, ‘oocytes’, ‘oxidative stress’, ‘mitochondrial dysfunction’, ‘DNA methylation’, ‘histone modifications’, ‘RNA’, ‘non-coding RNAs’ and ‘cell death pathways’. Reference lists of relevant articles and reviews were additionally screened to identify publications not retrieved during the initial database search.

Priority was given to original human studies directly examining cryopreservation-associated structural, functional, molecular or epigenomic changes. Where human mechanistic evidence was limited or unavailable, relevant mammalian experimental studies were included to provide biological context and identify potential mechanisms requiring validation in humans. Review articles were used primarily to provide background information, identify the relevant primary literature and place individual findings within the broader field of reproductive cryobiology. Publications unrelated to gamete cryopreservation, studies without relevance to the molecular or cellular mechanisms considered in this review, non-peer-reviewed material and publications for which sufficient methodological or biological information could not be evaluated were not considered central evidence.

Because the review was intended to provide a mechanistic and integrative narrative synthesis rather than an exhaustive quantitative assessment of all available studies, formal systematic-review procedures such as prospective protocol registration, PRISMA-based study selection, risk-of-bias scoring and meta-analysis were not applied. Accordingly, no claim of systematic-review methodology is made. Particular attention was instead given to distinguishing evidence obtained directly from human gametes from observations derived from animal or other experimental models and to identifying areas in which mechanistic evidence remains preliminary or requires confirmation in human studies.

## 3. Spermatozoa Cryopreservation (Freezing and Thawing)

Cryopreservation represents a well-established strategy for the long-term storage of human spermatozoa and the creation of sperm banks, enabling preservation of male fertility potential for future clinical use. This approach is particularly relevant for:

(1) Patients undergoing chemotherapy and/or radiotherapy for malignant diseases;
(2) Adolescents scheduled for gonadotoxic treatments for autoimmune disorders;
(3) Individuals with severe oligoasthenoteratozoospermia (OAT) or azoospermia;
(4) Men unable to provide a fresh semen sample on the day of assisted reproductive technology (ART) procedures;
(5) Healthy sperm donors [6].

Although human sperm cryopreservation has been practised for nearly eight decades and has undergone continuous technical refinement, spermatozoa remain highly susceptible to cryoinjury. Freezing and thawing are consistently associated with quantitative sperm loss and qualitative impairment, affecting motility, viability, membrane integrity and DNA stability. These alterations arise from a combination of biophysical and molecular stressors, including intracellular and extracellular ice formation, osmotic imbalance, oxidative stress and abrupt temperature transitions from physiological conditions to −196 °C and back to normothermia. Collectively, these processes are referred to as cryodamage and thaw damage.

### 3.1. Mechanisms of Cryodamage During Freezing

**Figure 1:**
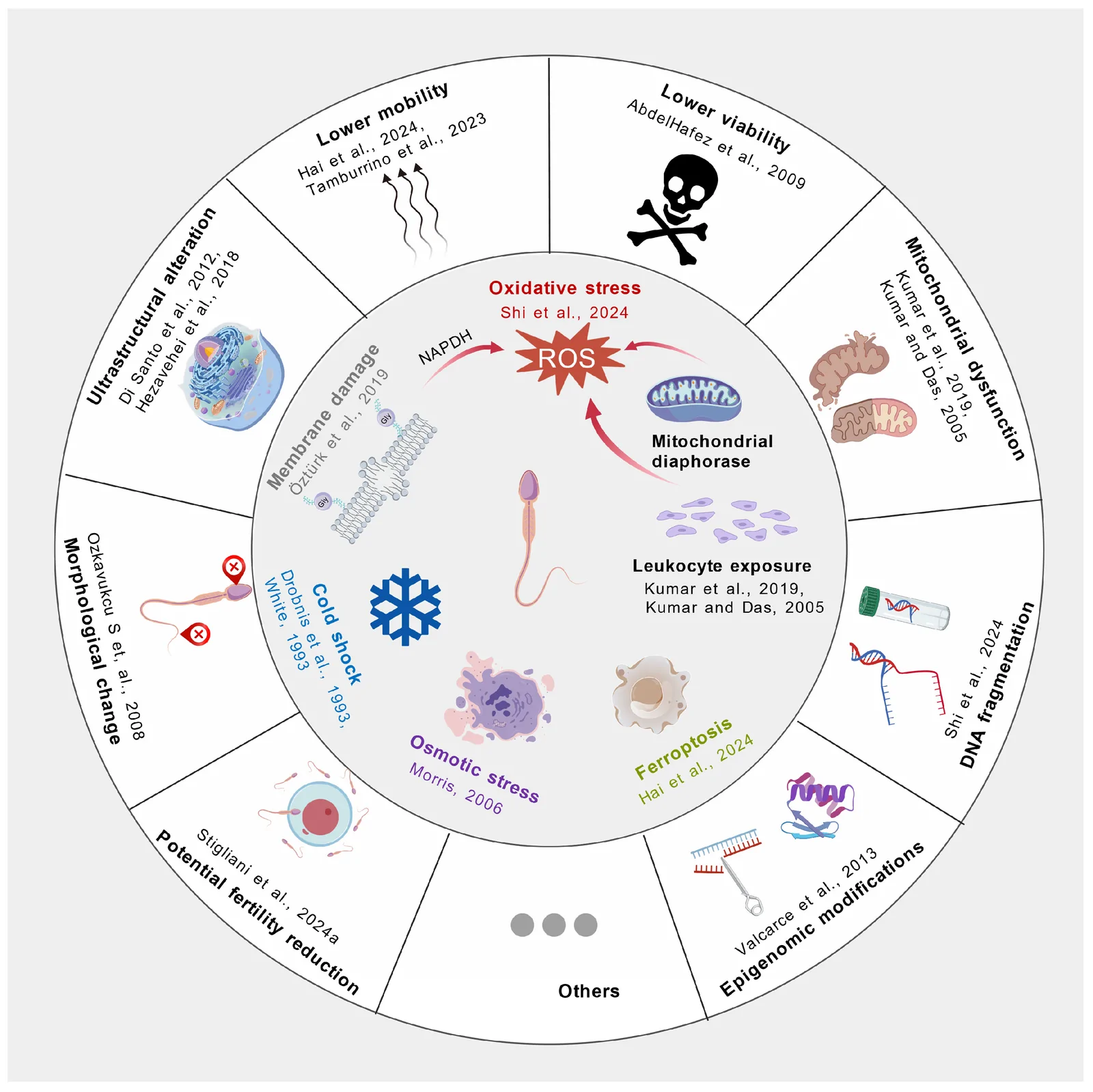
Cryopreservation-induced injury mechanisms in human spermatozoa ultimately impairing sperm viability, motility, and fertilization potential. Ozkavukcu et al., 2008 [7], Di Santo et al., 2012 [8], Hezavehei et al., 2018 [9], Drobnis et al., 1993 [10], White, 1993 [11], Morris, 2006 [12], Tamburrino et al., 2023 [13], Shi et al., 2024 [14], Kumar et al., 2019 [15], Kumar and Das, 2005 [16], Öztürk et al., 2019 [17], Valcarce et al., 2013 [18], Stigliani et al., 2024 [19], Hai et al., 2024 [20], AbdelHafez et al., 2009 [21], Stigliani et al., 2024 [22]. Figure 1 was generated using BioGDP.com [23].

The principal mechanisms underlying sperm cryodamage and their downstream consequences are summarized in Figure 1. Transition from physiological temperature to extreme hypothermia initiates a cascade of structural, biochemical and molecular disturbances in spermatozoa. Scanning electron microscopy analyses of human sperm after freezing and thawing have demonstrated increased frequencies of loose sperm heads, flagellar defects, acrosomal alterations and subacrosomal swelling (Ozkavukcu et al., 2008 [7], Figure 1).

Key cryopreservation-associated stressors: membrane phase transitions, osmotic imbalance, ice crystal formation, oxidative injury, mitochondrial dysfunction, DNA damage, and activation of regulated cell death pathways.

### 3.2. Membrane Alterations

The integrity of the sperm plasma membrane, which is rich in phospholipids and cholesterol, is critically affected during cooling from 36 °C to 5 °C and subsequently to −196 °C. Temperature-dependent phase transitions within the lipid bilayer disrupt lipid–protein interactions, altering membrane fluidity, ion transport and metabolic activity [17]. Importantly, post-thaw membrane reorganization is often incomplete, resulting in persistent defects in osmotic regulation and, in severe cases, cell death.

In addition, the sperm glycocalyx—a carbohydrate-rich outer layer composed primarily of glycoproteins and glycolipids—is markedly altered during cryopreservation (Di Santo et al., 2012 [8]; Hezavehei et al., 2018 [9], Figure 1). As the glycocalyx plays a central role in immune protection within the female reproductive tract, acrosome reaction and early sperm–oocyte interactions, its disruption compromises fertilization competence. Non-bilayer lipid phase transitions further contribute to cold shock injury [10,11]. Phosphatidylcholine has been identified as a protective lipid component, with spermatozoa enriched in phosphatidylcholine exhibiting superior post-thaw motility [24].

The relationship between glycerol and sperm cryoinjury is concentration- and exposure-dependent and reflects the balance between its protective and potentially detrimental effects. As a permeating cryoprotectant, glycerol reduces the amount of freezable intracellular water, lowers the probability of intracellular ice formation and modifies the physicochemical properties of the extracellular and intracellular solutions during cooling. However, its addition produces a rapid increase in extracellular osmolality, initially driving water out of the spermatozoon and causing cellular shrinkage. As glycerol subsequently permeates the plasma membrane, intracellular osmotic equilibrium is progressively restored and cell volume partially recovers. The reverse sequence occurs during glycerol removal after warming: extracellular glycerol concentration decreases more rapidly than intracellular glycerol concentration, promoting water influx and transient cellular swelling. Thus, spermatozoa may undergo repeated shrinkage–swelling cycles during cryoprotectant addition, freezing, warming and cryoprotectant removal. If these volume excursions exceed the osmotic tolerance of the sperm membrane, irreversible membrane destabilization, altered permeability, acrosomal injury and loss of functional competence may occur [6,9,12].

The protective effect of glycerol therefore cannot be separated from its potential osmotic and chemical toxicity. Increasing cryoprotectant concentration may improve suppression of ice formation, but simultaneously increases solution osmolality and the magnitude of cellular volume changes. Prolonged exposure may additionally perturb membrane lipid organization, membrane-associated proteins and cellular metabolism. Consequently, the effectiveness of glycerol depends not only on its final concentration but also on temperature, exposure time, rate of addition and removal, cooling rate and the composition of the accompanying non-permeating cryoprotective components. This explains why gradual addition and removal of glycerol are generally preferable to abrupt exposure to highly hyperosmotic cryoprotective solutions [8,9,12].

Other permeating cryoprotectants, including dimethyl sulfoxide (DMSO), ethylene glycol and propylene glycol, operate according to the same general principle of reducing freezable water and limiting intracellular ice formation, but they differ substantially in membrane permeability, molecular size, toxicity and the magnitude and duration of the osmotic volume excursions that they induce. DMSO penetrates biological membranes efficiently and provides strong cryoprotection against ice formation; however, its cytotoxicity is strongly dependent on concentration, temperature and exposure duration. Ethylene glycol has a relatively low molecular mass and high membrane permeability and may therefore permit more rapid equilibration and smaller transient changes in cell volume under appropriately controlled conditions. Nevertheless, no permeating cryoprotectant is intrinsically non-toxic, and increasing permeability does not eliminate the risks associated with excessive concentration or inappropriate loading and unloading [6,8,12].

Permeating cryoprotectants are frequently combined with non-permeating components, such as sucrose, trehalose, proteins or other macromolecular constituents. These extracellular agents do not provide intracellular protection in the same manner as glycerol, DMSO or ethylene glycol; instead, they contribute to controlled cellular dehydration, stabilization of membranes and reduction in the amount of water available for ice formation. The final cryoprotective effect therefore results from an interaction among CPA permeability, extracellular osmolality, membrane tolerance, cooling and warming kinetics and exposure time. For spermatozoa, which possess a highly specialized plasma membrane and limited capacity to tolerate extreme volume excursions, optimization of this balance is particularly important [6,8,9,12].

### 3.3. Ice Formation and Osmotic Stress

During freezing, extracellular ice crystals form first, leading to a progressive increase in extracellular solute concentration and driving water efflux from the sperm cell. The rate of cooling influences intracellular ice formation; however, ultrastructural studies indicate that sperm damage correlates more strongly with osmotic stress induced by glycerol than with cooling rate per se [13]. Rapid cellular dehydration results in cell shrinkage and mechanical disruption of both the acrosomal and plasma membranes, thereby impairing sperm function.

### 3.4. Oxidative and Molecular Damage

Structural destabilization of spermatozoa is accompanied by profound molecular injury, including DNA fragmentation, mitochondrial membrane depolarisation, protein loss and excessive generation of reactive oxygen species (ROS). Oxidative stress has been identified as a dominant contributor to sperm cryodamage (Shi et al., 2024 [14], Figure 1). Major sources of ROS include:

(1) NADPH oxidase activity at the sperm plasma membrane;
(2) Mitochondrial NADH-dependent oxidoreductase (diaphorase);
(3) Contaminating leukocytes within the semen sample (Kumar et al., 2019 [15]; Kumar and Das, 2005 [16], Figure 1).

At the genomic and transcriptomic levels, cryopreservation has been associated with damage to genes involved in fertilization competence and early embryonic development. Notably, SNORD116/PWSAS and UBE3A—paternally expressed genes implicated in Prader–Willi and Angelman syndromes—were shown to be particularly vulnerable following vitrification (Valcarce et al., 2013 [18], Figure 1). Whole-transcriptome analyses further revealed 219 downregulated and 28 upregulated transcripts after one week of conventional cryostorage, affecting pathways related to spermatogenesis, sperm motility, mitochondrial function, calcium homeostasis and early embryo development (Stigliani et al., 2024 [19], Figure 1). Integrated transcriptomic, proteomic and metabolomic profiling has consistently highlighted mitochondrial dysfunction as a hallmark of cryoinjury, with alterations in the tricarboxylic acid cycle and oxidative phosphorylation pathways. Among the most consistently affected genes was mitochondrial NAD(P)+ transhydrogenase [25].

### 3.5. Cell Death Pathways

Sperm cryodamage encompasses both accidental cell death and regulated cell death (RCD), triggered respectively by intracellular ice formation and oxidative stress-mediated signalling cascades (Hai et al., 2024 [20], Figure 1).

Cryopreservation-induced sperm cell death is mechanistically heterogeneous and should not be attributed to a single dominant pathway. Depending on the magnitude and nature of the cryogenic insult, spermatozoa may exhibit features associated with apoptosis, necrotic cell death and ferroptosis. These mechanisms are not necessarily mutually exclusive within a cryopreserved sperm population, and different subpopulations may undergo different forms of injury according to their initial physiological condition, membrane composition, antioxidant capacity and severity of exposure to thermal, osmotic and oxidative stress.

Apoptosis is a regulated process involving mitochondrial and/or receptor-mediated signalling and downstream activation of proteolytic pathways. In cryopreserved spermatozoa, mitochondrial membrane depolarization, oxidative stress and membrane perturbation may promote apoptosis-associated changes, including phosphatidylserine externalization, alterations in mitochondrial function and activation of caspase-dependent signalling. In contrast, severe physicochemical injury may result in loss of membrane integrity, ionic homeostasis and cellular structural organization, producing necrotic or necrosis-like cell death. Such injury may occur when extreme osmotic stress, intracellular ice formation or mechanical membrane disruption exceeds the capacity of the spermatozoon to maintain cellular homeostasis.

Ferroptosis represents a mechanistically distinct form of regulated cell death characterized primarily by iron-dependent accumulation of lipid peroxides rather than by the canonical caspase cascade associated with apoptosis. This mechanism is particularly relevant to sperm biology because sperm membranes contain high levels of polyunsaturated fatty acids and are therefore intrinsically susceptible to lipid peroxidation. Cryopreservation-induced oxidative imbalance may increase the generation of reactive oxygen species while simultaneously reducing antioxidant protection, thereby facilitating oxidative damage to membrane phospholipids. In the presence of redox-active iron, propagation of lipid peroxidation can progressively compromise membrane structure and cellular function.

Importantly, identification of ferroptosis as a contributor to sperm cryoinjury does not imply that it is more frequent or quantitatively more important than apoptosis or necrotic cell death. Current evidence supports its consideration as an additional pathway that may operate alongside other forms of cryopreservation-induced injury. The relative contribution of apoptosis, necrosis and ferroptosis is likely to depend on the freezing protocol, cryoprotective conditions, oxidative environment and intrinsic characteristics of the sperm sample. Consequently, further comparative studies using pathway-specific molecular markers are required to determine their relative contributions to human sperm cryodamage [20,21,26].

Pharmacological inhibition of ferroptosis using α-lipoic acid has been shown to improve post-thaw sperm quality in both humans and goats (Hai et al., 2024 [20], Figure 1).

### 3.6. Cryotolerance Variability

The extent of cryodamage varies markedly between individuals and species and is influenced by intrinsic sperm characteristics, including fatty acid composition and the ω-3/ω-6 ratio (Hezavehei et al., 2018 [9], Figure 1). Spermatozoa from asthenozoospermic or oligoasthenozoospermic men are particularly susceptible to cryoinjury, resulting in reduced fertilization capacity. Cryosurvival is commonly expressed as the ratio of post-thaw to prefreeze motile spermatozoa [26]. Even in normozoospermic samples, 30–50% of sperm may lose motility or viability following cryopreservation, depending on freezing protocols, cryoprotectant formulations and individual susceptibility (Hai et al., 2024 [20]; Tamburrino et al., 2023 [13], Figure 1). A systematic review reported an overall mean post-thaw survival rate of 46.5%, with values ranging from 8% to 85% (AbdelHafez et al., 2009 [21], Figure 1).

Optimal outcomes are achieved when semen samples are processed within one hour of ejaculation, with initial motility ≥55% and minimal motility decline within the first four hours [27]. Importantly, long-term storage duration—up to a median of nine years—has not been shown to adversely affect key sperm physiological pathways or functional competence (Stigliani et al., 2024 [22], Figure 1).

### 3.7. Mechanisms of Cryodamage During Thawing

Thawing represents an inevitable and critical phase of sperm cryopreservation, during which cellular injury may be as severe as, or even exceed, that induced during freezing. The principal mechanisms underlying thaw-associated damage include ice recrystallisation, osmotic shock and lipid peroxidation (Di Santo et al., 2012 [8], Figure 1).

During warming, small ice crystals formed during freezing may coalesce into larger structures through recrystallisation, mechanically disrupting cellular membranes and organelles. Experimental supplementation with graphene oxide has been shown to reduce recrystallised ice grain size, thereby preserving post-thaw sperm structural integrity and function [28].

Temperature fluctuations during thawing induce additional phase transitions within the sperm plasma membrane. Phospholipid phase transitions occur at approximately 20 °C, while glycolipid transitions take place around 33 °C, resulting in membrane destabilization and osmotic imbalance [29]. Accordingly, optimization of thawing temperature significantly influences functional recovery. Thawing at 40 °C, rather than the conventional 37 °C, has been associated with an approximately 23.1% increase in sperm motility [30].

The rate of warming constitutes another key determinant of post-thaw sperm quality. Direct immersion of frozen sperm pellets into thawing medium at 37 °C for 20 min resulted in higher viability, reduced acrosomal damage and lower chromatin condensation compared with a two-step thawing protocol involving initial exposure to room temperature followed by warming at 37 °C [31]. However, these observations remain controversial, as other studies have reported no significant differences between rapid and slow thawing strategies [32], highlighting the continued lack of consensus regarding optimal warming protocols.

In addition, semen volume has been identified as a contributory factor influencing post-thaw recovery. A thawing volume of 0.5 mL has been shown to confer superior outcomes compared with 0.25 mL, including improved total and progressive motility, enhanced mitochondrial membrane potential and better preservation of chromatin integrity [33].

## 4. Human Oocyte Cryopreservation (Freezing and Thawing)

The late 1980s marked a major milestone in assisted reproduction with the first successful pregnancies achieved using cryopreserved human oocytes through slow-freezing and rapid-thawing techniques [34,35]. In contrast to male fertility, which remains relatively stable over decades, female reproductive potential declines sharply with age due to progressive depletion and deterioration of oocyte quality. Biologically, optimal female reproductive capacity is observed between approximately 15 and 30 years of age; however, social, educational and professional factors have increasingly led women to postpone childbearing [35].

Female reproductive potential progressively declines with advancing age, with the decline becoming more pronounced during the later reproductive years as both oocyte quantity and quality decrease [35].

Consequently, oocyte cryopreservation (OC) has emerged as a cornerstone strategy for fertility preservation across a broad range of clinical and non-clinical indications, including:

(1) Women undergoing chemotherapy and/or radiotherapy for malignant diseases;
(2) Patients at risk of premature ovarian insufficiency;
(3) Individuals with poor ovarian response employing oocyte accumulation strategies;
(4) Cycles in which no sperm sample is available on the day of oocyte retrieval;
(5) Healthy oocyte donors;
(6) Women electively delaying childbearing for social or personal reasons [36,37].

Similar to spermatozoa, human oocytes are exposed to multiple injurious processes during cryopreservation; however, their exceptionally large size, high water content and low surface area-to-volume ratio markedly exacerbate their susceptibility to cryodamage. These properties predispose oocytes to intracellular ice crystal formation, osmotic stress and thermal shock, leading to disruption of membrane integrity, altered intracellular–extracellular fluid balance and, ultimately, compromised viability.

**Figure 2:**
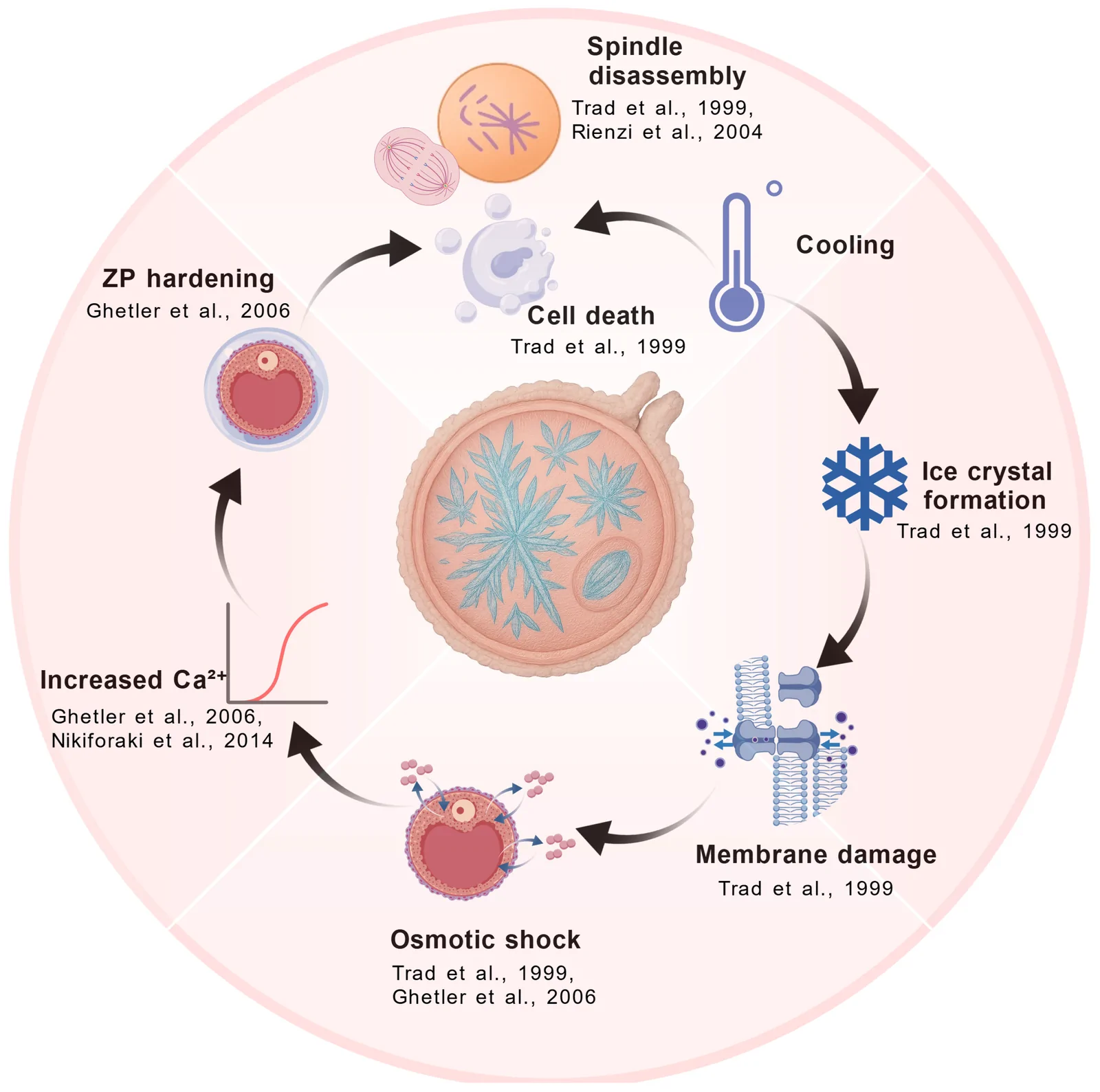
Cryopreservation-induced cellular injury and meiotic spindle disruption in human oocytes. Key cryopreservation-associated stressors: osmotic imbalance, ice crystal formation, oxidative injury, zona pellucida hardening, cytoskeletal instability, meiotic spindle depolymerization, and altered calcium signaling. (ROS) reactive oxygen species; (ZP) zona pellucida. Trad et al., 1999 [38], Rienzi et al., 2004 [39], Ghetler et al., 2006 [40], Nikiforaki et al., 2014 [41]. Figure 2 was generated using BioGDP.com [23].

Ice crystal formation remains a central challenge in mammalian oocyte cryopreservation. Cooling rate and temperature sensitivity critically influence oocyte survival, with marked inter-oocyte variability. Certain human oocytes are damaged at relatively high seeding temperatures (approximately −4.5 °C), whereas others tolerate lower temperatures (<−8 °C). Ice seeding at −8 °C results in substantial oocyte loss, while seeding at −6 °C improves outcomes but still adversely affects temperature-sensitive oocytes. To minimize intracellular ice formation while preserving viability, an optimal seeding temperature range of −4.0 to −4.5 °C has been proposed (Trad et al., 1999 [38], Figure 2).

Elevating extracellular ice seeding temperatures closer to the melting point facilitates controlled oocyte dehydration prior to reaching critical temperatures. A two-step cooling protocol (−0.2 °C/min to −15 °C followed by −1 °C/min) has been shown to achieve near-equilibrium dehydration while limiting solute toxicity (Trad et al., 1999 [38], Figure 2).

During thawing, previously immobilized water molecules regain mobility, promoting expansion of existing ice nuclei, formation of new crystalline structures and mass redistribution through recrystallization, whereby larger ice crystals grow at the expense of smaller ones [2]. Notably, thawing has been demonstrated to exert a greater impact on oocyte and embryo viability than cooling itself [37]. Cryoprotective agent (CPA) type and concentration further modulate ice dynamics: at low CPA concentrations, maximal ice formation during warming is largely independent of cooling rate, whereas at higher CPA levels it decreases with accelerated cooling. Reducing CPA concentration by ≥20% promotes ice crystallisation during cooling, increases heterogeneity of ice diffraction patterns and enlarges crystalline domains during warming [2].

During warming, ice structures formed during cooling may undergo recrystallization, a process in which smaller ice crystals disappear while larger crystals grow. Such crystal growth may increase the probability of mechanical and structural cellular injury. The extent of ice formation and recrystallisation is influenced by multiple interacting variables, including CPA type and concentration, cooling and warming rates, sample geometry and the physical properties of the biological system. In general, insufficient cryoprotectant concentration may permit greater ice formation, whereas adequate CPA exposure combined with appropriately controlled cooling and, particularly, warming conditions reduces the probability of damaging crystallisation. However, these relationships are strongly protocol- and model-dependent and should not be interpreted as universally applicable quantitative thresholds for human oocytes.

In clinical practice, most ART centres preferentially cryopreserve mature metaphase II (MII) oocytes, following first polar body extrusion and alignment of chromosomes at the meiotic spindle equatorial plate. At this stage, both nuclear and cytoplasmic maturation are largely complete. However, although rapid cooling preserves gross cellular architecture, it arrests developmental processes and may compromise genomic stability, particularly through perturbation of the meiotic spindle (MS) apparatus. The MS is a highly dynamic microtubule-based structure composed of α/β-tubulin heterodimers and microtubule-associated proteins, and is essential for accurate chromosome segregation during meiosis. Spindle integrity is critical for the generation of euploid haploid gametes and subsequent embryo development.

The MS begins to depolymerise at temperatures as high as 33 °C, with further destabilization at lower temperatures, and can fully disassemble within 10 min of exposure to non-physiological temperature or pH conditions (Trad et al., 1999 [38], Figure 2).

At the metaphase II stage, nuclear and cytoplasmic maturation are largely complete; however, the meiotic spindle remains a particularly temperature-sensitive cellular structure. Exposure to non-physiological temperatures during cryopreservation may induce spindle depolymerization, disorganization or altered chromosome–microtubule interactions. Such changes should not be interpreted as direct evidence that cryopreservation causes genomic instability. Rather, impaired spindle organization may interfere with accurate chromosome alignment and segregation after warming and could therefore increase the risk of chromosome-segregation errors if normal spindle architecture is not restored before fertilization. The biological relevance of spindle alterations consequently depends not only on their occurrence immediately after warming but also on their reversibility during post-warming recovery.

During slow-freezing, the proportion of oocytes exhibiting normal spindle configuration may decrease by up to 20% following one hour of incubation [42]. Advances in polarized light microscopy, combined with liquid crystal optics and sophisticated imaging software, now permit non-invasive real-time visualization of the MS in living MII oocytes.

Compared with conventional slow freezing, modern vitrification protocols may provide improved preservation of meiotic spindle architecture in mature human oocytes; however, spindle integrity after warming is not uniformly maintained under all experimental conditions. The extent of spindle preservation and subsequent recovery depends on multiple variables, including the vitrification system, CPA composition and exposure, cooling and warming rates, temperature control during handling, and the interval between warming and assessment. Transient spindle depolymerization or disorganization may therefore occur after vitrification, with partial or complete reassembly during subsequent incubation under appropriate conditions.

Accordingly, the presence or absence of an apparently normal spindle immediately after warming should not be considered a protocol-independent characteristic of vitrified oocytes. Rather, spindle recovery represents a dynamic process whose kinetics depend on the cryopreservation and warming procedure and on post-warming culture conditions. These considerations are clinically relevant because the timing of ICSI after warming should allow adequate oocyte recovery while simultaneously avoiding unnecessary prolongation of post-warming culture and oocyte ageing (Rienzi et al., 2004 [39], Figure 2, [43]).

Cryopreservation by either vitrification or slow freezing may also induce premature hardening of the zona pellucida (ZP), a critical extracellular matrix involved in sperm binding and prevention of polyspermy. This phenomenon is attributed to unphysiological cortical granule exocytosis, resulting in biochemical modification of ZP proteins and impaired fertilization. Consequently, ICSI has become the standard fertilization method for warmed oocytes, yielding superior outcomes compared with conventional IVF. Notably, both mature (MII) and immature (germinal vesicle, GV) oocytes exhibit a marked reduction in cortical granule density following cryopreservation, accompanied by vesicle formation within the ooplasm (Ghetler et al., 2006 [40], Figure 2).

Following cryopreservation and warming, fertilization rates reported for human oocytes vary considerably according to oocyte maturity, cryopreservation method, warming protocol, patient characteristics and the method used for insemination. Conventional IVF is particularly dependent on preservation of normal sperm–zona pellucida interaction and is therefore potentially compromised when cryopreservation induces zona pellucida hardening or premature cortical granule exocytosis. ICSI circumvents these barriers by direct introduction of a spermatozoon into the ooplasm and is consequently the preferred fertilization method for warmed human oocytes. Reported fertilization rates following ICSI of vitrified mature human oocytes are generally comparable to those obtained with fresh mature oocytes when survival after warming is satisfactory, whereas conventional IVF has historically shown lower and more variable fertilization efficiency following oocyte cryopreservation. Accordingly, fertilization rates should be interpreted in relation to the cryopreservation technique and clinical setting rather than as a single universal percentage.

Despite ongoing technical refinements, CPA exposure remains an unavoidable component of both slow-freezing and vitrification protocols. CPA concentration directly influences osmotic stress and promotes intracellular water efflux (dehydration) [44]. A major biophysical constraint of oocyte cryopreservation is the extremely low surface area-to-volume (SA:V) ratio of oocytes (approximately 0.05), compared with spermatozoa (approximately 4.3). This markedly reduced SA:V ratio slows osmotic equilibration during CPA exposure, necessitating longer equilibration periods and lower optimal cooling rates [44]. Osmotically induced volume fluctuations during CPA loading and unloading may result in membrane rupture or sublethal cellular injury, adversely affecting laboratory handling and clinical outcomes [45]. Accordingly, CPA introduction and removal must be performed gradually and in strict coordination with cooling and warming protocols.

Cryopreservation-associated osmotic stress and physiological fertilization-induced Ca^2+^ signalling should be considered distinct, although potentially interacting, processes. During CPA loading and removal, rapid changes in extracellular osmolality produce substantial oocyte volume changes and may perturb membrane permeability, ion transport and intracellular Ca^2+^ homeostasis. Experimental observations indicate that osmotic challenge can be associated with transient changes in intracellular Ca^2+^ concentration and may influence processes related to oocyte activation and cortical granule exocytosis. However, these stress-associated Ca^2+^ responses should not be interpreted as equivalent to the highly organized Ca^2+^ oscillations that normally occur following fertilization.

Under physiological conditions, sperm–oocyte fusion initiates a characteristic series of intracellular Ca^2+^ transients that coordinates oocyte activation, cortical granule exocytosis, completion of meiosis and progression toward pronuclear formation. Cryopreservation-related perturbation of Ca^2+^ homeostasis may potentially modify the ability of the warmed oocyte to generate or respond appropriately to this physiological signalling pattern. Importantly, however, evidence for individual components of this mechanism has been obtained under different experimental conditions, and not all proposed links between osmotic stress, Ca^2+^ elevation, cortical granule release and polar body extrusion have been demonstrated directly and sequentially in cryopreserved human oocytes.

Human studies have nevertheless provided evidence that oocyte cryopreservation and subsequent in vitro culture can modify Ca^2+^ signalling during fertilization. Accordingly, the available evidence supports an association between cryopreservation-related cellular stress and altered Ca^2+^ homeostasis, while the precise causal sequence and its contribution to subsequent fertilization competence remain incompletely defined (Ghetler et al., 2006 [40], Figure 2). Calcium signalling plays a pivotal role in fertilization: following sperm–oocyte fusion, sperm-derived phospholipase C zeta induces a prolonged initial Ca^2+^ transient lasting approximately five minutes, followed by repetitive oscillatory spikes that persist for several hours until pronuclear formation. These Ca^2+^ oscillations regulate cortical granule exocytosis, cell cycle progression, activation of maturation-promoting factor and mitogen-activated protein kinase, and recruitment of maternal mRNAs essential for early embryogenesis [42].

Importantly, altered Ca^2+^ oscillation patterns have been observed following slow cryopreservation and thawing (Nikiforaki et al., 2014 [41], Figure 2), which may partially account for the reduced fertilization rates reported for cryopreserved oocytes compared with fresh or in vitro-matured oocytes [46,47].

## 5. Epigenomic Molecular Alterations During Cryopreservation (Freezing and Thawing) of Human Gametes (Spermatozoa and Oocytes)

**Figure 3:**
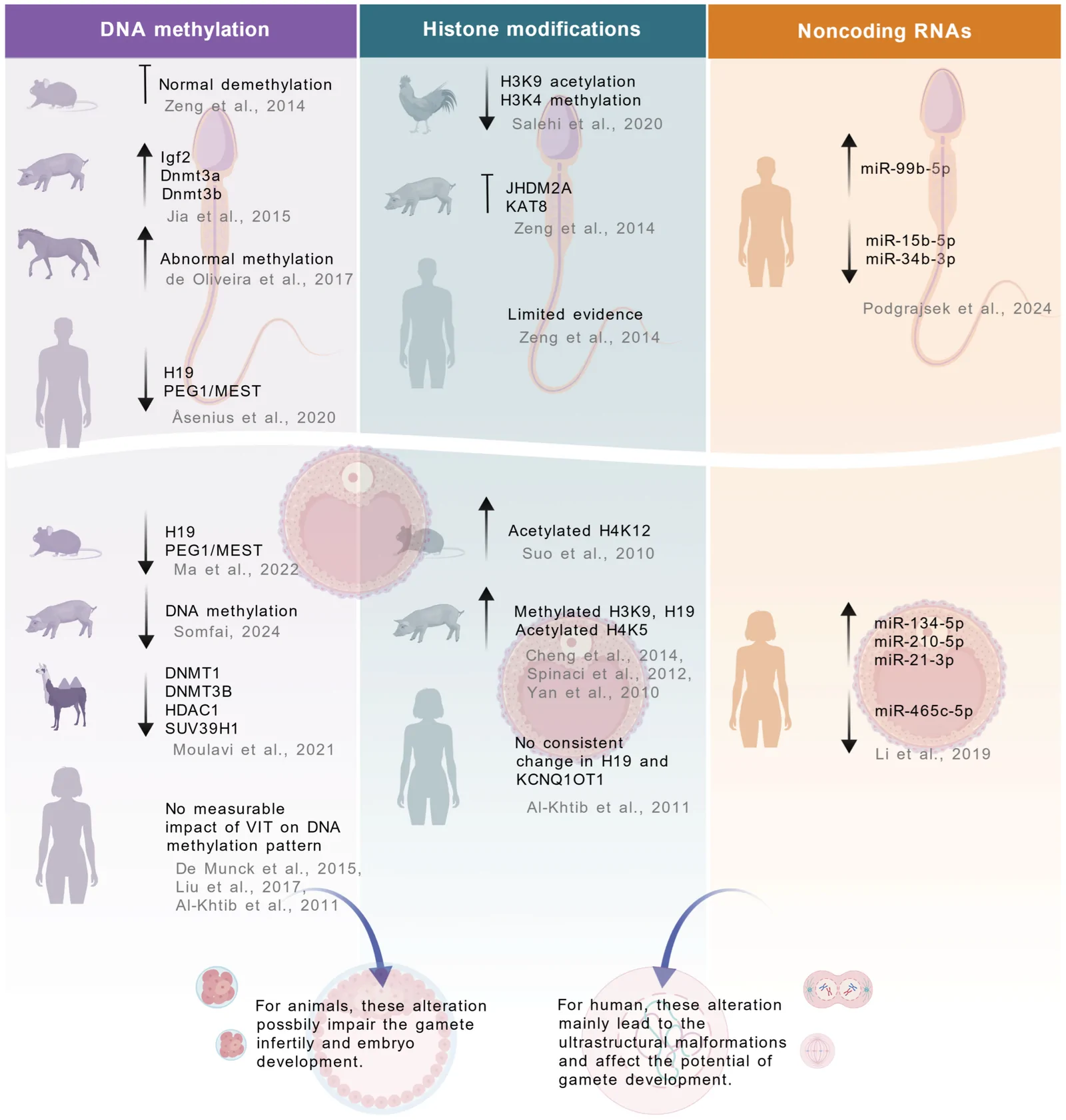
Cryopreservation-induced epigenomic alterations in gamete. Cryopreservation-induced changes in DNA methylation, histone modifications, and non-coding RNA expression in human spermatozoa and oocytes. Flat-ended downward arrows indicate disruption; upward arrows indicate increased expression or upregulation; downward arrows indicate decreased expression or downregulation. (VIT) vitrification. Suo et al., 2010 [5], Zeng et al., 2014 [49], Jia et al. [50], de Oliveira et al. [51], Åsenius et al., 2020 [52], Moulavi et al., 2021 [53], Ma et al., 2022 [54], Somfai, 2024 [55], Liu et al., 2017 [56], Al-Khtib et al., 2011 [57], Salehi et al., 2020 [58], Yan et al., 2010 [59], Spinaci et al., 2012 [60], Cheng et al., 2014 [61], Podgrajsek et al., 2024 [62], Li et al. [63], De Munck et al., 2015 [64]. Figure 3 was generated using BioGDP.com [23].

Cryopreservation has raised persistent concerns regarding its potential to induce epigenomic alterations in human gametes. The epigenome comprises heritable chemical modifications of DNA and chromatin that regulate gene expression without altering the underlying nucleotide sequence. Preservation of epigenomic integrity is essential for normal fertilization competence, embryonic genome activation, developmental programming and long-term offspring health. The principal epigenetic regulatory layers include DNA methylation, post-translational histone modifications and non-coding RNAs (ncRNAs) [48]. Disruption of any of these finely balanced regulatory systems during cryopreservation could, at least theoretically, compromise subsequent embryo development and developmental trajectory (Figure 3).

### 5.1. DNA Methylation Following Gamete Cryopreservation

DNA methylation is an important component of epigenomic regulation, but its functional consequences depend on genomic context rather than representing a universal mechanism of transcriptional repression. In particular, DNA methylation within promoter and regulatory regions is frequently associated with reduced transcriptional accessibility, whereas methylation within gene bodies and other genomic regions may have different functional relationships with gene expression. Beyond transcriptional regulation, DNA methylation plays essential roles in genomic imprinting, X-chromosome inactivation, maintenance of genome stability and regulation of repetitive and centromeric sequences. These context-dependent functions are particularly relevant to gametes, in which highly regulated establishment, maintenance and subsequent reprogramming of DNA methylation patterns are required for normal fertilization and early embryonic development [1].

Imprinted loci are of particular relevance, as they regulate parent-of-origin-specific gene expression patterns that extend well beyond the blastocyst stage and are critical for fetal growth and placental development.

A comprehensive review identified nine imprinting disorders reported in association with assisted reproductive technologies (ART), of which only three—Beckwith–Wiedemann syndrome, Angelman syndrome and maternal hypomethylation syndrome—have shown a possible association with ART [65]. Importantly, the authors emphasized that establishing causality remains challenging, as underlying subfertility itself may predispose to imprinting errors. Moreover, within ART cycles, cryopreservation represents only one of several potential epigenetic stressors; ovarian stimulation, IVF or ICSI procedures, in vitro culture conditions and parental health status may also independently influence epigenomic outcomes.

Evidence from animal models indicates that sperm cryopreservation may influence epigenetic regulation, although the reported effects differ substantially among species and experimental conditions. Zeng et al. [49] investigated epigenetic alterations associated with cryopreservation of boar spermatozoa (Figure 3), whereas Jia et al. [50] demonstrated that cryopreservation of mouse spermatozoa can affect pronuclear formation and DNA demethylation during early zygotic development.

However, not all preservation procedures are associated with detectable epigenetic changes. In equine semen, de Oliveira et al. [51] reported that cooled storage did not induce major alterations in sperm DNA methylation (Figure 3). Importantly, cooled semen storage and cryopreservation involving freezing and thawing represent different experimental conditions and should not be considered interchangeable.

These observations demonstrate that the molecular consequences of gamete preservation depend on the animal species, preservation method and biological endpoints investigated. Accordingly, findings obtained under one set of experimental conditions should not be directly extrapolated to other species or preservation protocols.

In contrast, human data are comparatively limited and largely reassuring. Two independent studies assessing frozen human spermatozoa—one analyzing samples stored for four weeks after swim-up preparation [66], and another following vitrification—reported no detectable changes in global DNA methylation levels, even when cryopreservation was associated with increased reactive oxygen species (ROS) generation or elevated DNA fragmentation indices [67]. Nevertheless, it is important to recognize that sperm abnormalities themselves may carry intrinsic epigenetic defects independent of freezing. For example, oligozoospermia has been associated with hypomethylation at imprinted loci such as H19 and PEG1/MEST (Åsenius et al., 2020 [52], Figure 3).

Comparable observations have been reported in oocyte studies. In animal models, oocyte or embryo cryopreservation has been linked to altered expression of DNA methylation-related genes, including downregulation of DNMT1 (DNA Methyltransferase 1), DNMT3B (DNA Methyltransferase 3 Beta), HDAC1 (Histone Deacetylase 1), and SUV39H1 (Suppressor of Variegation 3–9 Homolog 1) in camel embryos (Moulavi et al., 2021 [53], Figure 3), reduced Dnmt1 and Kcnq1ot1 expression in mouse oocytes (Ma et al., 2022 [54], Figure 3), and decreased expression of methylation-associated genes in porcine models, potentially attributable to cryoprotectant exposure (Somfai, 2024 [55], Figure 3). These genes encode key components of the molecular machinery responsible for establishing and maintaining epigenomic organization. DNMT1 encodes DNA methyltransferase 1, an enzyme principally involved in maintenance of established DNA methylation patterns during DNA replication and therefore in preservation of epigenetic information. DNMT3B encodes DNA methyltransferase 3 beta, which participates predominantly in de novo establishment of DNA methylation patterns. Appropriate regulation of both enzymes is important for maintenance and reprogramming of DNA methylation during gametogenesis, fertilization and early embryonic development.

Altered expression of these enzymes may therefore disturb the balance between maintenance and establishment of methylation marks and potentially influence genomic imprinting and developmental gene regulation.

HDAC1 encodes histone deacetylase 1, an enzyme that removes acetyl groups from histone proteins and thereby contributes to chromatin compaction and regulation of transcriptional accessibility. Changes in HDAC1 expression may alter histone acetylation patterns and consequently modify chromatin organization and gene regulatory states. SUV39H1 encodes a histone methyltransferase involved particularly in methylation of histone H3 at lysine 9, a modification associated with formation and maintenance of transcriptionally repressive heterochromatin. Altered SUV39H1 activity may therefore affect chromatin condensation, genome stability and appropriate epigenetic regulation.

In the context of gamete fertility, these enzymes should not be considered isolated molecular markers but rather interacting components of the epigenetic machinery. Disturbance of their expression during or after cryopreservation could theoretically influence DNA methylation, chromatin accessibility and transcriptional regulation, with possible downstream consequences for gamete competence and early embryonic development. However, changes in gene expression do not necessarily demonstrate persistent functional epigenetic damage, and observations obtained in animal gametes or embryos should not be directly extrapolated to human reproductive outcomes. This distinction is particularly important because, as discussed above, available human data remain considerably more reassuring than some findings obtained in experimental animal models.

By contrast, human oocyte studies employing global epigenomic profiling (Liu et al., 2017 [56], Figure 3) or targeted analysis of key imprinted loci such as H19 and KCNQ1OT1 (Al-Khtib et al., 2011 [57], Figure 3) have consistently failed to detect significant alterations in DNA methylation patterns following vitrification. Collectively, these findings suggest that, while cryopreservation can perturb DNA methylation pathways in animal models, current clinical protocols do not appear to induce detectable methylation defects in human gametes.

### 5.2. Histone Modifications and Gamete Cryopreservation

Evidence that sperm cryopreservation alters histone modifications is currently derived predominantly from non-human experimental models. In boar spermatozoa, cryopreservation has been associated with altered expression of histone-modifying enzymes, including JHDM2A and KAT8, together with changes in chromatin organization and sperm function. Similarly, studies in rooster sperm have reported reductions in specific histone acetylation and methylation marks following freezing, accompanied by increased DNA fragmentation. These observations provide mechanistic evidence that cryopreservation can influence histone-associated epigenetic regulation in animal spermatozoa.

However, these findings should not be directly extrapolated to human spermatozoa. Species differ substantially in sperm chromatin organization, the proportion and genomic distribution of retained histones, membrane composition, cryotolerance and the cryopreservation protocols routinely applied. At present, direct evidence demonstrating comparable cryopreservation-induced changes in specific histone modifications in human spermatozoa remains limited. Animal studies should therefore be regarded primarily as mechanistic models identifying potentially cryosensitive epigenetic pathways that require confirmation in appropriately designed human studies [49,58].

Experimental studies in non-human species indicate that cryopreservation can disrupt histone modification pathways. In boar spermatozoa, cryopreservation was shown to alter the expression of histone-modifying enzymes, including JHDM2A and KAT8, leading to aberrant chromatin condensation, impaired gene silencing and compromised sperm function, with potential consequences for fertilization and early embryonic development (Zeng et al., 2014 [49], Figure 3). In rooster sperm, a marked reduction in H3K9 acetylation and H3K4 methylation was observed following freezing, accompanied by increased DNA fragmentation (Salehi et al., 2020 [58], Figure 3).

It was represented a substantial knowledge gap in human reproductive epigenetics (Zeng et al., 2014 [49], Figure 3).

Oocyte studies across species further support the sensitivity of histone marks to cryopreservation. In mouse oocytes, increased acetylation of histone H4 at lysine 12 (H4K12ac) after freezing was associated with defective chromatin condensation, elevated aneuploidy rates and reduced developmental competence (Al-Khtib et al., 2011 [57], Figure 3), suggesting a greater epigenomic resilience in the clinical setting.

### 5.3. Non-Coding RNAs and Gamete Cryopreservation

A substantial proportion of the human transcriptome consists of non-coding RNA (ncRNA) species that do not serve as conventional templates for protein synthesis but participate in diverse regulatory and structural processes. The estimated proportion of the genome undergoing transcription varies considerably according to the experimental methodology, transcription threshold and definition of functional RNA; therefore, a single quantitative estimate may be misleading. Functionally, ncRNAs comprise a heterogeneous group that includes housekeeping and structural RNAs, such as ribosomal RNAs (rRNAs), transfer RNAs (tRNAs), small nuclear RNAs (snRNAs) and small nucleolar RNAs (snoRNAs), as well as regulatory RNA classes including microRNAs (miRNAs), small interfering RNAs (siRNAs), Piwi-interacting RNAs (piRNAs) and long non-coding RNAs (lncRNAs) [59,60,61,68].

Emerging evidence indicates that cryopreservation can alter ncRNA expression profiles in human gametes. In spermatozoa, differential miRNA expression has been reported following freezing, with distinct patterns observed between normozoospermic and oligozoospermic samples. In particular, vitrification appeared to induce broader miRNA dysregulation than slow programmable freezing, which predominantly affected samples with normal semen parameters (Podgrajsek et al., 2024 [62], Figure 3). Notably, miR-99b-5p—associated with favourable Day-3 embryo development—was significantly upregulated in oligozoospermic samples after vitrification. Conversely, miR-15b-5p, implicated in early embryonic development, and miR-34b-3p, a regulator of spermatogenesis and germ cell development, were downregulated following slow freezing (Podgrajsek et al., 2024 [62], Figure 3). Prolonged storage duration further amplified cryopreservation-associated miRNA alterations [69].

Experimental evidence from mouse oocytes further suggests that vitrification may influence the expression of selected non-coding RNAs. Li et al. [63] reported differential expression of 22 miRNAs among 520 investigated candidates following oocyte vitrification (Figure 3). These findings indicate that cryopreservation-associated molecular stress may affect posttranscriptional regulatory mechanisms in experimental oocyte models.

However, these observations were obtained in mouse oocytes and should not be interpreted as direct evidence of comparable miRNA alterations in human oocytes. Differences in oocyte physiology, maturation and cryotolerance must be considered when evaluating the potential relevance of these experimental findings to human reproductive medicine.

Collectively, these data suggest that ncRNAs represent a sensitive molecular interface between cryopreservation-induced stress and downstream functional competence of gametes. However, the long-term developmental and clinical significance of these ncRNA alterations remains incompletely understood and warrants further investigation.

## 6. Discussion, Current Limitations and Future Perspectives

Gamete cryopreservation has become an indispensable component of modern reproductive medicine, providing effective fertility preservation for both medical and elective indications. Nevertheless, exposure of spermatozoa and oocytes to extreme thermal and osmotic stress during freezing and thawing inevitably induces structural, functional and molecular perturbations, including membrane destabilization, osmotic imbalance, oxidative injury and cytoskeletal disruption. Oocytes appear particularly vulnerable due to their large size, high water content and complex intracellular organization, whereas spermatozoa exhibit marked inter-individual variability in cryotolerance. Although animal studies suggest that cryopreservation may induce epigenomic alterations, available human data remain largely reassuring, with no consistent evidence for persistent changes in key imprinting marks or adverse developmental outcomes.

Looking forward, the field of gamete cryobiology is expected to transition from empirically optimized protocols towards molecularly informed strategies. Integration of multi-omics approaches—including transcriptomics, proteomics and metabolomics—will facilitate the identification of cryosensitive molecular signatures and enable the development of personalized cryopreservation protocols tailored to the biochemical and structural properties of individual gametes. In parallel, advances in artificial intelligence and biophysical modelling are likely to refine predictions of freezing and warming dynamics, allowing automated selection of optimal cooling and thawing trajectories.

At a practical level, continued progress will depend on the development of next-generation cryoprotectants that combine low cytotoxicity, high permeability and precise osmotic control. Promising approaches include bio-inspired antifreeze proteins, zwitterionic compounds and nanomaterial-based cryoprotectant carriers. In addition, technical innovations such as controlled ice nucleation, microfluidic vitrification platforms and ultra-rapid warming technologies hold significant potential to reduce ice recrystallisation and further improve post-thaw gamete viability.

From a translational perspective, convergence of cryobiology with regenerative medicine may enable long-term preservation of bioengineered ovarian and testicular tissues, opening new avenues for fertility restoration and oncofertility care. Finally, the expanding application of gamete cryopreservation—particularly in the context of planned or “social” freezing—underscores the need for ongoing multidisciplinary dialogue addressing ethical, societal and regulatory considerations.

The evidence reviewed here demonstrates that cryopreservation-induced injury cannot be adequately explained by a single molecular pathway. Rather, cryoinjury represents a multilevel process initiated by physical changes in temperature, water distribution, solute concentration and ice formation and subsequently propagated through membrane destabilization, osmotic imbalance, mitochondrial dysfunction, oxidative stress and alterations in cellular macromolecules. These processes are strongly interconnected. For example, membrane damage may disturb ionic homeostasis and mitochondrial function, whereas mitochondrial dysfunction may enhance ROS production, which in turn promotes lipid peroxidation, protein modification and DNA damage. Consequently, individual molecular alterations observed after warming should be interpreted as components of an interconnected cryoinjury network rather than as isolated events.

An important conclusion emerging from the available literature is that the strength of evidence differs considerably between individual mechanisms and experimental systems. In human spermatozoa, reductions in motility and viability and alterations in membrane integrity, mitochondrial function, oxidative balance and DNA/chromatin stability are comparatively well documented. By contrast, the relative contribution of individual regulated cell death pathways remains less clearly established. Ferroptosis represents an interesting emerging mechanism because of the particular susceptibility of sperm membrane polyunsaturated fatty acids to lipid peroxidation; however, its identification as a component of cryoinjury should not be interpreted as evidence that ferroptosis represents the predominant form of sperm cell death. Apoptotic, necrotic and ferroptotic processes may coexist, and their relative importance is likely to vary according to sperm quality, cryopreservation protocol and the magnitude of oxidative and osmotic stress.

The relationship between mitochondrial dysfunction, oxidative stress and epigenomic alterations deserves particular consideration. Cryopreservation-associated mitochondrial impairment may increase ROS generation and disturb cellular redox homeostasis, thereby promoting oxidative damage to DNA, membrane lipids and proteins. Such disturbances may also influence the stability of epigenetic information through oxidative modification of DNA, altered activity of chromatin-associated regulatory enzymes or changes in the abundance and stability of sperm-borne non-coding RNAs. These mechanisms provide a plausible molecular connection between the cellular injury described in Section 3 and the epigenomic alterations discussed in Section 5.

Nevertheless, the coexistence of mitochondrial dysfunction, oxidative stress and altered epigenetic markers does not establish a direct causal relationship. In particular, mature spermatozoa possess highly condensed chromatin and very limited transcriptional activity. Consequently, molecular differences detected after thawing may reflect selective survival of sperm subpopulations, changes in RNA stability or pre-existing epigenetic heterogeneity rather than newly induced and persistent epigenomic modifications. Establishing causality will require experimental studies that simultaneously assess mitochondrial function, oxidative injury, epigenomic characteristics and subsequent reproductive outcomes.

A similar distinction is required when interpreting epigenomic evidence. Animal models provide valuable mechanistic information and permit experimental approaches that are difficult or ethically impossible to perform in human gametes. They have demonstrated that cryopreservation can be accompanied by alterations in DNA methylation, histone-associated regulation and non-coding RNA expression. Nevertheless, species-specific differences in gametogenesis, chromatin organization, cryotolerance and cryopreservation protocols limit direct extrapolation of these findings to humans. Human studies remain fewer, frequently involve relatively limited sample sizes and often examine selected molecular endpoints rather than comprehensive epigenomic profiles. Importantly, the available human evidence has not demonstrated a consistent pattern of persistent cryopreservation-induced abnormalities at major imprinting-associated loci. Thus, experimental evidence of molecular change should be distinguished from evidence of clinically relevant epigenetic dysfunction.

This distinction is particularly important because demonstration of a molecular alteration immediately after warming does not necessarily establish a persistent biological consequence. Some cryopreservation-associated changes may represent transient cellular responses, selective survival of particular gamete subpopulations or reversible disturbances rather than permanent molecular injury. Conversely, the absence of a detectable global change does not exclude locus-specific or cell-specific alterations. Interpretation therefore depends strongly on the analytical method, timing of assessment, biological material examined and functional endpoint used. Future studies should increasingly combine molecular measurements with functional outcomes rather than considering statistically detectable molecular differences as synonymous with impaired fertility.

The comparison between spermatozoa and oocytes further illustrates the importance of cellular context. Both gamete types experience thermal, osmotic and oxidative stress, but their structural and functional vulnerabilities differ substantially. Spermatozoa possess a highly specialized plasma membrane, condensed chromatin and limited cytoplasmic volume, and post-thaw damage is particularly evident in motility, membrane and acrosomal integrity, mitochondrial activity and chromatin stability. Oocytes, in contrast, must preserve a large cytoplasmic compartment, meiotic spindle organization, cortical granule distribution, intracellular organelles and calcium-dependent activation mechanisms. Thus, apparently similar upstream cryogenic stress may produce different downstream biological consequences in male and female gametes.

Another important consideration is the balance between cryoprotection and cryoprotectant-associated injury. Modern cryopreservation protocols substantially reduce damage that would otherwise result from uncontrolled ice formation; however, complete elimination of cryoinjury is biologically unrealistic. Increasing cryoprotectant concentration may improve suppression of ice formation while simultaneously increasing chemical and osmotic stress. Similarly, faster cooling may favor vitrification under appropriate conditions but cannot compensate for inadequate cryoprotectant equilibration or warming. Optimization therefore requires simultaneous consideration of CPA permeability, concentration, exposure duration, cellular water transport and cooling and warming kinetics.

Apparently inconsistent findings among cryopreservation studies may arise from differences in biological susceptibility as well as experimental design. Species-specific characteristics of sperm plasma membrane composition, cholesterol distribution, chromatin organization and antioxidant capacity can substantially modify responses to thermal, osmotic and oxidative stress. Moreover, cryopreservation protocols differ in cryoprotectant composition and concentration, equilibration time, cooling and warming rates, and post-thaw handling. These variables may influence not only the magnitude of cellular injury but also the molecular pathways predominantly affected.

Differences in analytical methodology represent an additional source of variability. Global DNA methylation measurements, locus-specific methylation analyses, transcriptomic profiling and assessments of individual epigenetic regulators examine different biological endpoints and therefore may produce apparently divergent results. Furthermore, changes detected immediately after warming cannot necessarily be equated with persistent alterations during fertilization or embryonic development. Consequently, discrepancies among studies should be interpreted in relation to species, preservation protocols, analytical approaches and the timing of molecular assessment rather than automatically regarded as contradictory evidence. 

The increasing use of gamete and embryo cryopreservation in assisted reproductive technology raises important questions regarding the potential consequences of cryopreservation-associated molecular alterations for subsequent embryonic development and offspring health. These questions are particularly relevant because contemporary ART frequently involves combinations of ovarian stimulation, gamete manipulation, fertilization procedures, embryo culture and cryopreservation. Consequently, the biological effects of cryopreservation must be considered within the broader context of ART rather than as an isolated intervention.

From a mechanistic perspective, cryopreservation-associated damage to sperm DNA, chromatin organization or epigenetic regulatory mechanisms could potentially influence events occurring after fertilization. Similarly, disturbances of oocyte mitochondrial function, meiotic spindle organization or intracellular calcium signalling may affect fertilization competence and early embryonic development. However, the detection of such alterations in gametes does not necessarily imply that they persist after fertilization or produce clinically relevant consequences in offspring.

Epigenetic regulation represents a particular area of interest because DNA methylation, histone-associated chromatin organization and regulatory RNAs participate in early developmental programming. Experimental studies in animal models have demonstrated that cryopreservation may be associated with changes in selected epigenetic markers. Nevertheless, the persistence of these changes during embryogenesis and their potential consequences for offspring development remain incompletely characterized. Species-specific differences in epigenetic reprogramming further limit direct extrapolation of animal findings to humans.

Available human studies investigating selected imprinting-associated methylation patterns have not consistently demonstrated persistent abnormalities attributable specifically to gamete cryopreservation. However, these observations should be interpreted cautiously because many studies involve limited sample sizes, restricted molecular endpoints and heterogeneous cryopreservation protocols. Furthermore, clinical outcomes following ART may be influenced by parental age, underlying infertility, ovarian stimulation, embryo culture conditions and other treatment-related factors, making it difficult to isolate the independent contribution of cryopreservation.

Accordingly, current molecular evidence does not establish a direct causal relationship between cryopreservation-induced epigenomic alterations in human gametes and adverse long-term offspring outcomes. At the same time, the available evidence is insufficient to exclude all possible subtle or delayed developmental effects. Further investigations integrating gamete molecular profiling, embryo development, pregnancy outcomes and long-term offspring follow-up are therefore necessary to clarify the clinical significance of cryopreservation-associated molecular changes.

Cryopreservation represents a well-established strategy for the long-term storage of human spermatozoa and the preservation of male reproductive potential. Its principal clinical applications include fertility preservation before gonadotoxic chemotherapy or radiotherapy, preservation in patients with severe impairment of spermatogenesis, storage of surgically retrieved or otherwise limited sperm samples, donor sperm banking, and situations in which a fresh semen sample may not be available at the time of assisted reproductive treatment [6,8,9,21]. Ice formation during oocyte cryopreservation is strongly influenced by cooling conditions, cryoprotectant exposure and the biophysical characteristics of the oocyte. In human oocytes subjected to slow-freezing protocols, experimental observations have demonstrated marked sensitivity to ice-seeding temperature, with survival depending on the balance between controlled extracellular ice formation, progressive cellular dehydration and avoidance of intracellular ice formation. Importantly, these observations were obtained under specific experimental slow-cooling conditions and should not be generalized to all contemporary oocyte cryopreservation protocols, particularly vitrification.

More broadly, cryobiological studies indicate that cooling and warming rates interact with cryoprotectant concentration to determine the probability of ice nucleation, crystal growth and recrystallisation. However, the magnitude and biological consequences of these processes depend strongly on the experimental system, cell type, CPA composition and thermal protocol. Therefore, mechanistic observations derived from model solutions, animal gametes or embryos provide important physicochemical information but should be distinguished from evidence obtained directly in human oocytes. In the present review, findings from human oocytes are consequently identified as such, whereas evidence derived from other experimental systems is used primarily to explain general cryobiological mechanisms.

Gamete cryopreservation has become an indispensable component of modern reproductive medicine, providing effective fertility-preservation options across a broad range of medical and non-medical indications. Nevertheless, exposure to low temperatures, osmotic gradients, cryoprotectants and cooling–warming transitions can induce structural, functional and molecular alterations in spermatozoa and oocytes. The magnitude of these changes is not uniform and depends on multiple factors, including gamete type and quality, cryopreservation method, CPA composition, cooling and warming kinetics and individual biological variability.

In human spermatozoa, the most consistently documented post-thaw alterations involve reduced motility and viability, membrane and acrosomal disruption, oxidative imbalance, mitochondrial dysfunction and increased susceptibility to DNA/chromatin damage. In human oocytes, reported cryopreservation-associated changes include osmotic and membrane stress, alterations in meiotic spindle organization, cortical granule distribution and Ca^2+^ signalling. Importantly, many of these alterations may be transient or partially reversible, and their detection immediately after warming does not necessarily indicate persistent functional impairment.

The evidence concerning epigenomic effects requires particularly cautious interpretation. Animal and experimental models demonstrate that cryopreservation can be associated with changes in DNA methylation, histone modifications and ncRNA expression. Direct human evidence is substantially more limited and heterogeneous. Available studies of human gametes have not identified a consistent pattern of persistent cryopreservation-induced abnormalities at the major imprinting-associated loci investigated to date, while changes in selected ncRNAs and other molecular endpoints have been reported. However, the number and size of human studies remain limited. The molecular endpoints and cryopreservation protocols are heterogeneous, and comprehensive longitudinal epigenomic data are scarce. 

Consequently, the absence of a consistent signal in currently available human studies should not be interpreted as evidence that all potential epigenomic effects have been excluded. In particular, evidence linking cryopreservation-associated molecular changes in human gametes to persistent alterations during embryonic development or to long-term health outcomes remains insufficient. Conversely, molecular alterations observed in experimental or animal models should not be assumed to produce clinically meaningful effects in humans. Future studies integrating molecular analyses with fertilization, embryo development and long-term reproductive and developmental outcomes will therefore be necessary to establish the biological and clinical significance of these observations.

## Data Availability

No new data were created or analyzed in this study. Data sharing is not applicable to this article.
